# EGFR-PI3K-PDK1 pathway regulates YAP signaling in hepatocellular carcinoma: the mechanism and its implications in targeted therapy

**DOI:** 10.1038/s41419-018-0302-x

**Published:** 2018-02-15

**Authors:** Hongwei Xia, Xinyu Dai, Huangfei Yu, Sheng Zhou, Zhenghai Fan, Guoqing Wei, Qiulin Tang, Qiyong Gong, Feng Bi

**Affiliations:** 1 Laboratory of Molecular Targeted Therapy in Oncology, State Key Laboratory of Biotherapy, West China Hospital of Sichuan University, 610041 Chengdu, Sichuan Province China; 2 Department of Medical Oncology and Cancer Center, West China Hospital of Sichuan University, 610041 Chengdu, Sichuan Province China; 3 Department of Radiology, West China Hospital of Sichuan University, 610041 Chengdu, Sichuan Province China

## Abstract

The epidermal growth factor receptor (EGFR) pathway and Hippo signaling play an important role in the carcinogenesis of hepatocellular carcinoma (HCC). However, the crosstalk between these two pathways and its implications in targeted therapy remains unclear. We found that the activated EGFR signaling could bypass RhoA to promote the expression of YAP(Yes-associated protein), the core effector of the Hippo signaling, and its downstream target Cyr61. Further studies indicated that EGFR signaling mainly acted through the PI3K-PDK1 (Phosphoinositide 3-kinase-Phosphoinositide-dependent kinase-1) pathway to activate YAP, but not the AKT and MAPK pathways. While YAP knockdown hardly affected the EGFR signaling. In addition, EGF could promote the proliferation of HCC cells in a YAP-independent manner. Combined targeting of YAP and EGFR signaling by simvastatin and the EGFR signaling inhibitors, including the EGFR tyrosine kinase inhibitor (TKI) gefitinib, the RAF inhibitor sorafenib and the MEK inhibitor trametinib, presented strong synergistic cytotoxicities in HCC cells. Therefore, the EGFR-PI3K-PDK1 pathway could activate the YAP signaling, and the activated EGFR signaling could promote the HCC cell growth in a YAP-independent manner. Combined use of FDA-approved inhibitors to simultaneously target YAP and EGFR signaling presented several promising therapeutic approaches for HCC treatment.

## Introduction

Hepatocellular carcinoma (HCC), a major malignancy of the liver, is the fifth most common cancer and the third leading cause of cancer-related mortality worldwide. HCC has a poor prognosis, only 15–20% of patients could survive more than 5 years^1^. Although some targeted therapies, such as sorafenib, can improve the clinical outcome, their effects are limited^2–4^. So there is a great need for more complete understanding of the molecular mechanisms involved in HCC development, which could help us design some new or improve therapeutic strategies for this illness.

Hippo pathway is an evolutionarily conserved mechanism that restricts organ size from drosophila to mammals. The core upstream components of this pathway comprise several tumor suppressors, including Mst1/2, Sav1/WW45, Lats1/2, and Mob1, which act in a kinase cascade that culminate in the phosphorylation and inactivation of YAP/TAZ (transcriptional co-activator with PDZ-binding motif). YAP/TAZ could act as the transcriptional co-activators to promote the expression of their target genes involved in proliferation and survival. Many studies have implicated that Hippo signaling pathway played a vital role in the tumorigenesis of HCC^5,6^. Conditional over-expression of YAP in transgenic mice or the liver-specific knockout of Mst1/2 or Sav1 could lead to expanded liver size and ultimately induce HCC, and these are the direct evidences for the importance of the Hippo pathway in regulating organ size and liver tumorigenesis^7–11^. Moreover, many clinical studies have illustrated that over-expression and nuclear accumulation of YAP could also act as an independent prognostic marker for the overall survival and disease-free survival in HCC patients, as well as in several other tumor types^12–14^. RhoA, a small G protein that belongs to the Rho family of Ras GTPase superfamily, plays a vital role in the regulation of many biological activities including actin organization, cell motility, proliferation, apoptosis and development^15^. Several recent studies indicated that RhoA participated in the activation of YAP through inducing stress fiber formation, and the G-protein-coupled receptor (GPCR) signaling could act through RhoA to regulate the Hippo-YAP pathway^16,17^.

Epidermal growth factor receptor (EGFR) signaling also plays an important role in hepatocellular carcinogenesis^3,18^. Several studies have indicated that EGFR was frequently over-expressed and positively correlated with early tumor recurrence in HCC. So anti-EGFR might be a promising therapeutic strategies in HCC^19–21^. Intriguingly, anti-EGFR therapy has achieved a huge success in both of lung cancer and colorectal cancer. However, it failed in HCC and the mechanisms behind remained elusive^3,18^. Recent reports indicated that the crosstalk between EGFR signaling and Hippo pathway was involved in the carcinogenesis in several other cancers^13,22–24^. However, the crosstalk between these two pathways and its implications in targeted therapy remain unclear in HCC^25^.

Here, we found that the EGF/EGFR signaling could bypass RhoA, a canonical YAP regulator, to activate YAP signaling in HCC cells^26^. Further investigation indicated that EGF mainly acted through PI3K/PDK1 pathway, but not the AKT and the MAPK pathway, to regulate YAP signaling. Our study also showed that EGF could promote cell proliferation in a YAP-independent manner. Importantly, our novel therapeutic strategies by simultaneously targeting EGFR signaling and YAP with combined use of the FDA-approved drugs demonstrated synergistic cytotoxicities in HCC cells.

## Results

### The expression of EGFR and YAP in human HCC cells

**Fig. 1:**
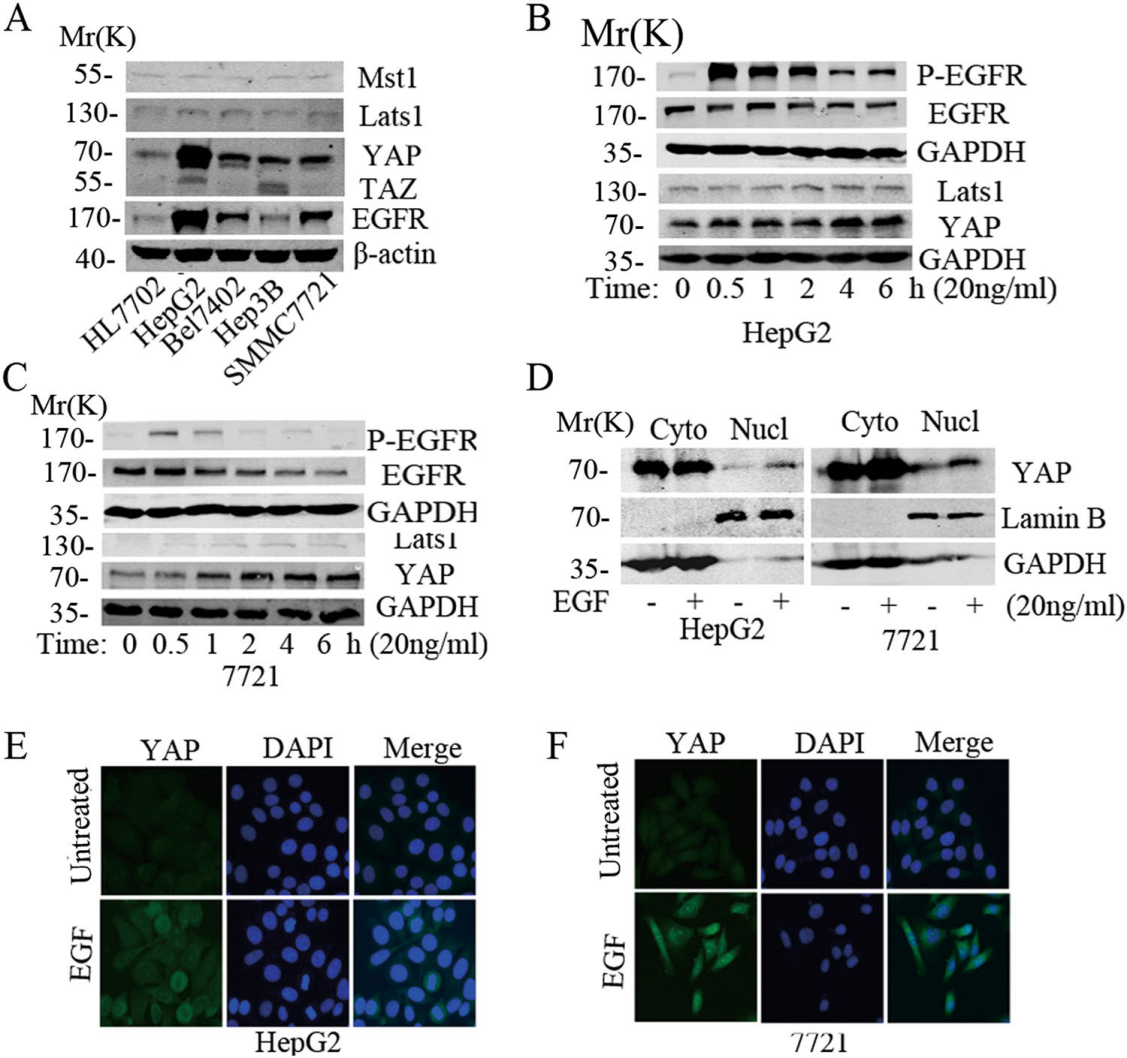
The activated EGFR signaling enhanced the expression of the core Hippo signaling effector YAP. **a** WB was used to examine the expression of EGFR and the core effectors of Hippo signaling in HCC cells. **b**, **c** Time dependent effect of EGF on the expression YAP and Lats1 in HepG2 and SMMC 7721 cells. **d** The HepG2 and 7721 cells were plated in the 10 cm dish and then serum-free starved for one night. After 20 ng/ml EGF stimulated for 2 h, the cytoplasmic and nuclear proteins were extracted. The protein samples were subjected to immunobloting with the indicated antibodies. GAPDH and LaminB were as cytoplasm and nucleus loading control, respectively. **e**, **f** Immunofluorescence was used to detect the effect of 20 ng/ml EGF stimulation on the expression and localization of YAP in HepG2 and SMMC7721 cells

To better understand the role of EGFR and Hippo signaling in HCC, western blot (WB) was performed to examine the expression of EGFR and the core members of Hippo signaling, Wnt signaling and Rho in HCC cell lines (Fig. 1a, Suppl. Fig. 1). We verified that EGFR and YAP were simultaneously up-regulated in all the 4 cancer cell lines compared with the normal liver cell HL7702, and only the expression of YAP was positively correlated with EGFR in these cells. In contrast, the expression of other Hippo signaling effectors, including Mst1, Lats1, and TAZ, had no obvious correlation with EGFR (Fig. 1a). We then selected two EGFR high-expressed cells for further investigation. Our studies indicated that activated EGFR signaling could enhance the expression of Lats1 and YAP in HepG2 and SMMC7721 cells in a time-dependent manner (Fig. 1b, c). Our investigation of the effect of EGF on the expression of the core members of EGFR signaling indicated that EGF treatment could stimulate the phosphoralation of EGFR, ERK and Akt1, and enhance the expression of CyclinD1 in HepG2 and 7721 cells (Fig. 1b, c, Suppl. Figs. 2A, B). Further cytoplasmic and nuclear protein extraction assays (Fig. 1d) and immunofluorescence study (Fig. 1e,f) indicated that EGF stimulation could promote the expression and nuclear accumulation of YAP in these two HCC cells.

### Activated EGFR signaling could enhance the expression of YAP in a RhoA-independent manner

**Fig. 2:**
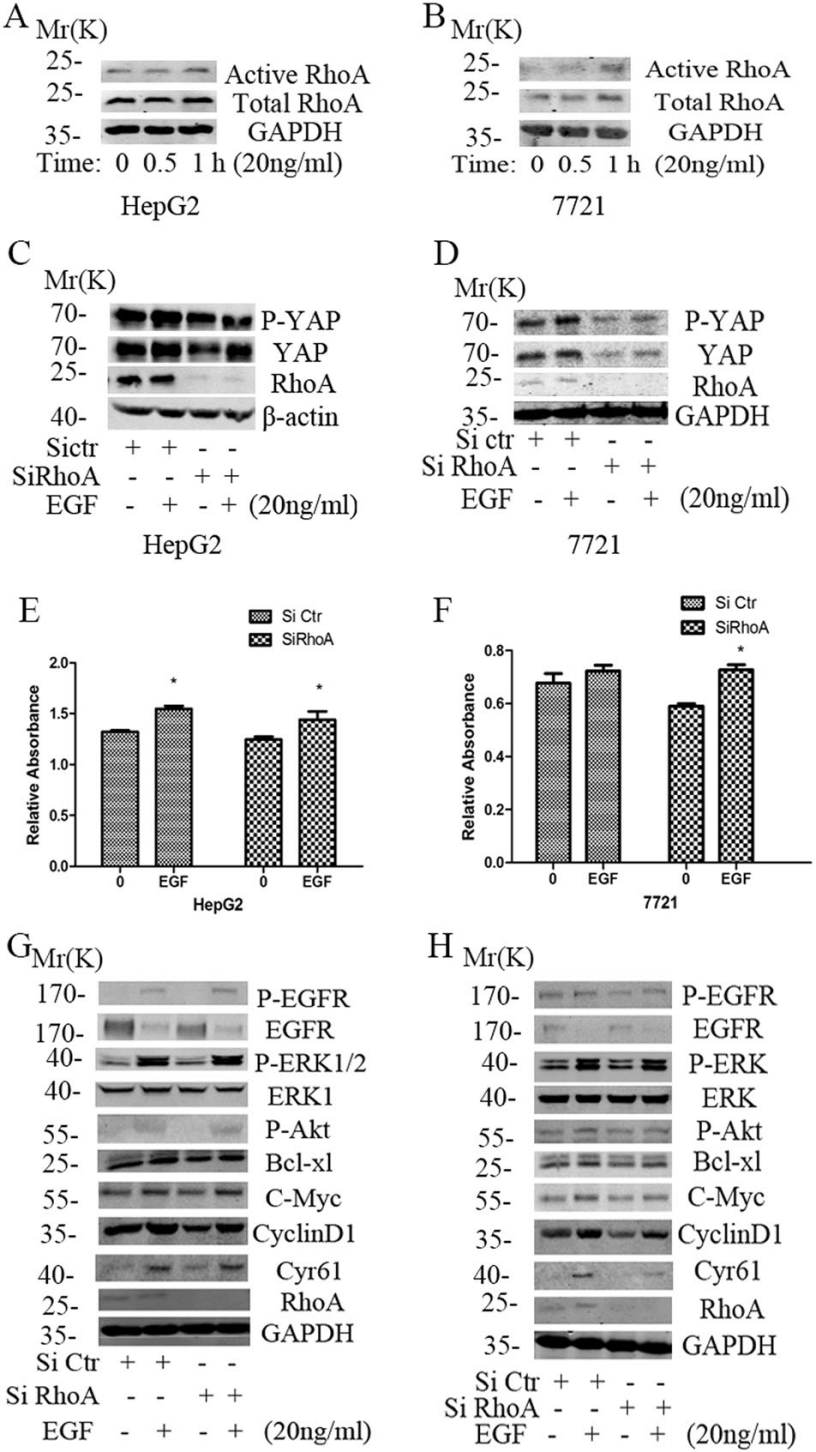
The activated EGFR signaling could enhance the expression of YAP in a RhoA independent manner in HCC cells. **a**, **b** Rhotekin pulldown assay was performed to examine the effect of 20 ng/ml EGF on the activity of RhoA in HepG2 and SMMC7721 cells. **c**, **d** After serum starving for one night, western blot were performed to detect the effect of 20 ng/ml EGF on the expressions of YAP in the cells transfected with SiRhoA in HepG2 and SMMC7721 cells. **e,** **f** CCK8 assays were used to detect the effect of RhoA knockdown combined with 50 ng/ml EGF stimulation for 48 h on the proliferation of the two HCC cell lines. **g,** **h** WB was used to examine the effect of combined treatment on the core effectors of the EGFR downstream signaling

Several recent studies have indicated that RhoA played a vital role in G protein coupled receptor (GPCR) mediated YAP activation^26^, while some reports showed that EGF could enhance the activity of RhoA^27^. Therefore, we tested whether EGFR signaling could act through RhoA to regulate YAP in HCC. The results revealed that EGF could up-regulate the activity and expression of total RhoA in the HepG2 and 7721 cell lines (Fig. 2a, b, Suppl. Fig. 2). Knockdown RhoA could partly inhibit the expression of YAP in HepG2 cells, and significantly reduce the expression of YAP in 7721 cells. However, EGF treatment could still stimulate the expression of YAP in the two HCC cells with RhoA knockdown (Fig. 2c, d). And the treatment had nearly no effect on its phosphorylation level (P-YAP/YAP) (Suppl. Fig. 3).

Previous data suggested a critical role of RhoA in EGF-mediated carcinogenesis^27^, so we also investigated whether the EGFR signaling could act via RhoA to promote the malignant phenotype of HCC cells. The results indicated that knockdown RhoA could partly inhibit cell proliferation, while EGF treatment could still promote proliferation of the HCC cells with RhoA knockdown (Fig. 2e, f). Further mechanism studies revealed that RhoA knockdown had nearly no effect on the EGFR signaling, but could partly inhibit the expression of C-Myc and CyclinD1. EGF treatment could still activate the EGFR signaling and Cyr61, the main downstream target of YAP, and partly enhance the expression of C-Myc, CyclinD1 and Bcl-xl in HepG2 and 7721 cells (Fig. 2g, h). Together, these results suggested that the activated EGFR signaling could bypass RhoA to activate YAP and promote cell proliferation in HCC cells.

### EGF mainly acts through the EGFR-PI3K-PDK1 pathway to regulate YAP in HCC cells

**Fig. 3:**
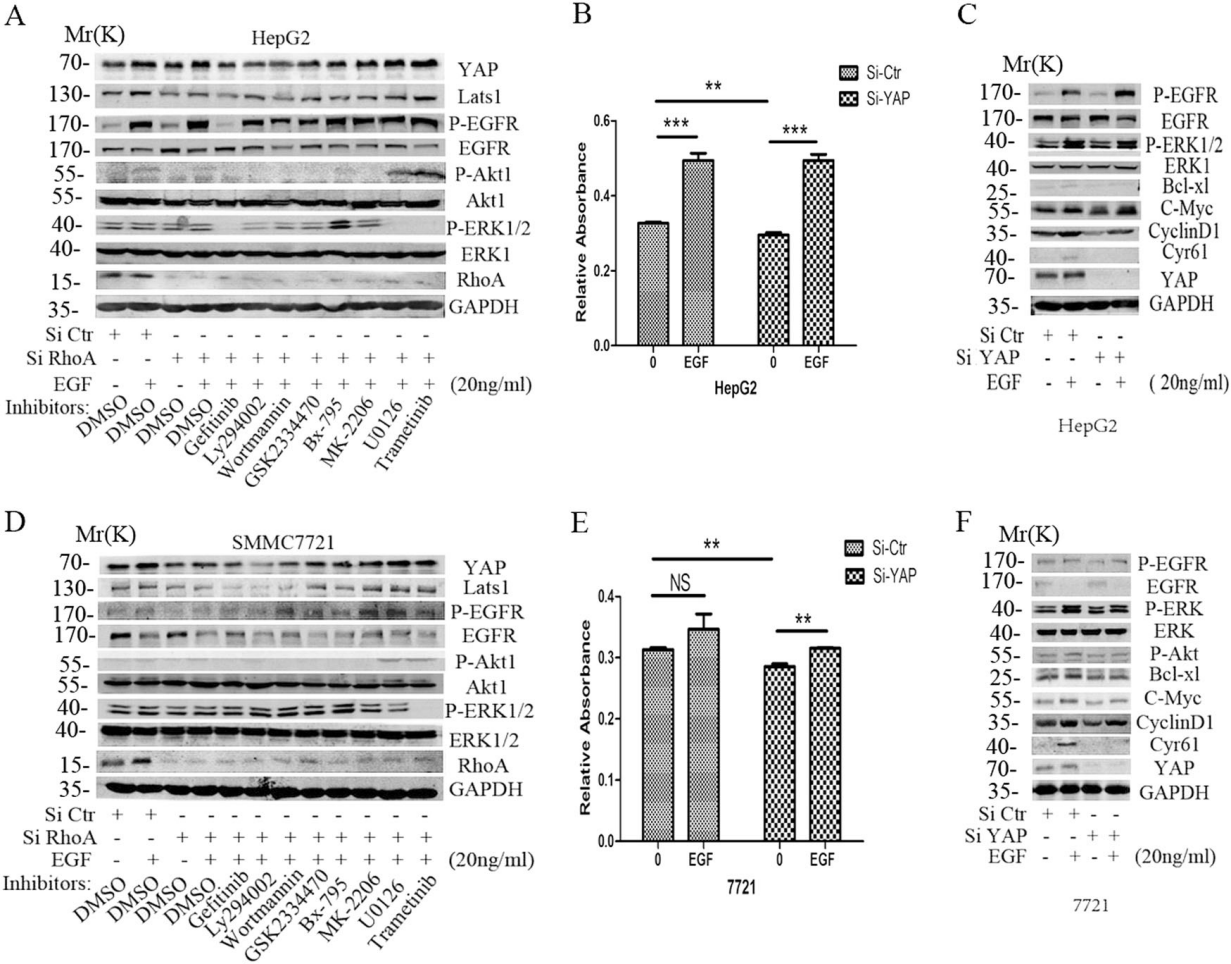
EGF mainly acts through the EGFR-PI3K-PDK1 pathway to enhance the expression of YAP in HCC cells. **a, b** WB was used to detect the effect of EGF treatment for 4 h on the expression of YAP with the inhibitors of EGFR or its downsream members, including the EGFR inhibitor (10uM Gefitinib), PI3K inhibitor (10 uM LY294002, 5 uM Wortmannin), PDK1 inhibitor (10uM GSK2334470, 10 uM BX-795), pan-Akt inhibitor(10 uM MK-2206) and MEK inhibitor(10 uM Trametinib, 10 uM U0126) in the Si RhoA transfected HepG2 and SMMC7721 cells for 48 h in HepG2 and SMMC7721 cells. **c, d** CCK8 assays were used to detect the effect of YAP knockdown combined with 50 ng/ml EGF stimulation for 48 h on the proliferation of the two HCC cell lines. **e,** **f** WB was used to examine the effect of combined treatment on the core effectors of the EGFR downstream signaling. *T*-test was used to detect the difference. (*P < 0.05, **P < 0.01, ***P < 0.001)

To explore the mechanism that involved in EGF induced YAP up-regulation, we used specific pharmacologic inhibitors to examine which pathway mainly mediated YAP activation in HCC cells. The results indicated that the inhibitors of PI3K(LY294002 and Wortmannin) significantly blocked EGF-mediated YAP over-expression as effectively as the EGFR inhibitor gefitinib. Inhibitors of the PI3K downstream effector PDK1 (GSK2334470 and BX-795) were also able to block YAP enhancement, while inhibitors of another major kinases, including AKT and MEK, had nearly no effect on the expression of YAP (Fig. 3a, d, Suppl. Fig. 4). The present data indicated that EGF mainly acted through the EGFR-PI3K-PDK1 pathway to regulate YAP in HCC cells.

### Activated EGFR signaling promotes the proliferation of HCC cells in a YAP independent manner

Since YAP plays an important role in the carcinogenesis of HCC^5^, we examined whether activated EGFR signaling could act through YAP to regulate the malignant phenotype of HCC cells. YAP siRNA was used to knock-down YAP protein in HepG2 and 7721 cells, while non-targeting siRNA was used as a control. Western blot analysis demonstrated that YAP siRNA successfully reduced YAP protein level in the two HCC cells (Fig. 3c, f). Knockdown YAP could significantly inhibit cell proliferation compared with the non-targeting controls. However, EGF could still promote the proliferation of HCC cells with YAP knockdown (Fig. 3b, e). Further WB results showed that knockdown YAP could inhibit the expression of Cyr61, one of the YAP target genes, but had nearly no effect on the phoshorylation of EGFR and its downstream effector P-ERK and P-AKT. Knockdown YAP could reverse the EGF induced Cyr61 over-expression in HCC cells, indicating that EGF could act though YAP to regulate its downstream target genes of Cyr61. However, EGF treatment could still activate EGFR and its downstream effectors, AKT and ERK, and the expression of C-Myc, CyclinD1and Bcl-xl in HepG2 and SMMC7721 cells with YAP knockdown (Fig. 3c, f). In all, these data indicated that the activated EGFR signaling could bypass YAP to promote the proliferation of HCC cells.

Therefore, taking into consideration that both YAP and EGFR signaling play a vital role in the carcinogenesis of HCC, combined targeting the Hippo and EGFR signaling pathways might provide a novel therapeutic strategy for HCC treatment.

### Anti-neoplastic potency of simvastatin in combination with the EGFR-TK inhibitor gefitinib

Several pharmacological inhibitors, including verteporfin and statins, have been reported to inhibit the function of YAP in tumorigenesis. Verteporfin could disrupt the YAP-TEAD interaction, however, it has been mainly used to eliminate abnormal blood vessels in the eye^28^. Statins, primarily used for primary and secondary prevention of cardiovascular diseases worldwide, could inhibit YAP through inhibiting RhoA activity^29,30^. Several large population-based cohort studies in Asian and Western populations have also shown that statins could reduce the risk of hepatocellular carcinoma^31–33^. So we used simvastatin as a YAP inhibitor in this study (Suppl. Figs. 5, 6).

**Fig. 4:**
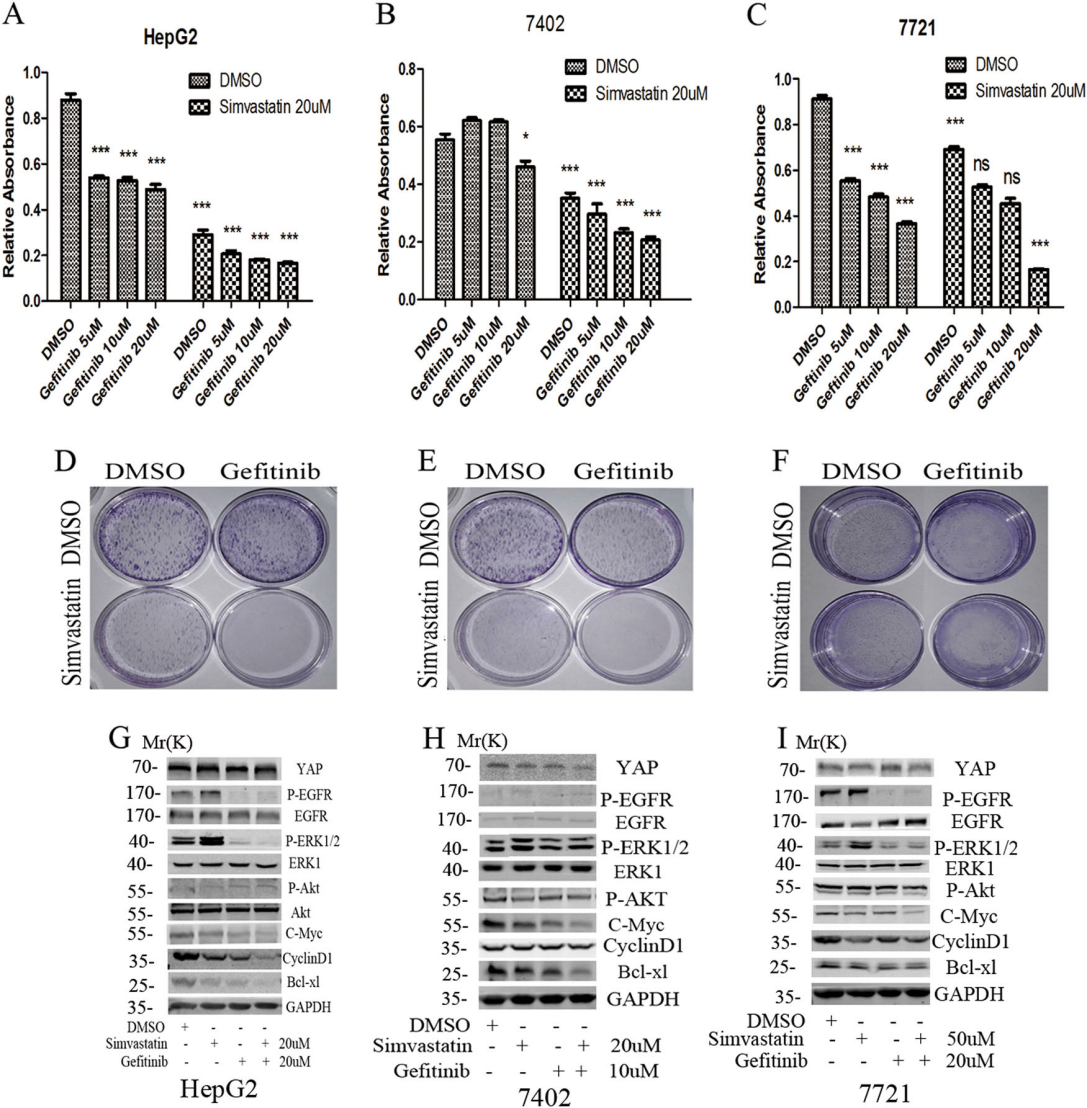
The combined effect of simvastatin and gefitinib in HCC cells. **a, b, c** CCK8 assays were used to detect the effect of simvastatin (YAP inhibitor) combined with gefitinib (EGFR inhibitor) on the proliferation in HepG2, Bel 7402 and SMMC7721 HCC cells. **d, e, f** Colony formation assays were used to detect the effect of simvastatin (YAP inhibitor) combined with gefitinib (EGFR inhibitor) on the survival of HCC cells. **g, h, i** WB was used to examine the effect of combined treatment on the core effectors of downstream signaling

To check the potency of simultaneous targeting EGFR signaling and Hippo pathway for more effective treatment, we firstly combined EGFR TKI gefitinib with YAP inhibitor simvastatin in HCC cells. The HCC cells were treated with simvastatin alone or in combination with rising concentrations of the gefitinib for 48 h. CCK8 assays showed that simvastatin treatment alone induced a strong growth inhibitory effect on the three HCC cell lines (HepG2, 7402, and 7721) after 48 h of continuous exposure to the drug. Gefitinib’s anti-neoplastic effect was significantly enhanced when the EGFR-TK inhibitor was combined with simvastatin for 48 h in those HepG2, 7402, and 7721cells (Fig. 4a, b, c, Suppl. Table 1). To investigate the coefficient of drug interaction (CDI), HCC cells were treated with a range of simvastatin and gefitinib, alone or in combination with a fixed concentration of ratio (1:1) in HepG2 and 7402 cells, and (2:1) in 7721 cells, after 48 h, cells viability was determined by Cell Counting Kit-8. Then we calculated the combination index (CI) values and the Dm using CompuSyn software (ComboSyn, Inc., Paramus, NJ, USA). According to the method proposed by Chou et al., combination index (CI) values <1, =1, >1, respectively, indicated synergistic, additive and antagonistic effect. The median effect dose (Dm) is the dose required to produce the median effect (analogous to the IC50). Linear regression correlation coefficients (*r*-values) of the median effect plots reflect that the dose-effect relationships for single treatment and the combination treatment, conform to the principle of mass action (in general, *r*-values >0.9 confirm the validity of this methodology). The Dm value for simvastatin, gefitinib, and the combination treatment was 8.140, 12.183, and 11.212 μM, respectively, in HepG2 cells, and a summary of the data from the same analysis applied to each of the five simvastatin/gefitinib combinations tested demonstrated that combinations exhibited synergistic therapeutic interactions (CI < 1) across a wide range (~0.55–1) of Fa values (Suppl. Table 2, Suppl. Fig. 10). The Dm value for simvastatin, gefitinib, and the combination treatment was 11.715, 53.759, and 5.962 μM, respectively, in 7402 cells, and the combinations exhibited synergistic therapeutic interactions across a wide range (~0.15–0.85) of Fa values (Suppl. Table 3, Suppl. Fig. 10). The Dm value for simvastatin, gefitinib, and the combination treatment was 65.086, 23.480, and 15.813 μM, respectively, in 7721 cells, and the combinations exhibited synergistic therapeutic interactions across a wide range (~0–0.55) of Fa values (Suppl. Table 4, Suppl. Fig. 10). Colony formation assays revealed that simvastatin combined with gefitinib inhibited the cell survival more significantly than that of simvastatin or gefitinib alone in HCC cells (Fig. 4d, e, f). We also studied the potential mechanism involved in the combined effect. WB results indicated that simvastatin alone could inhibit the expression of YAP, cyclinD1, C-Myc, and Bcl-xl, however, it had nearly no effect on the EGFR signaling, including P-EGFR, P-AKT, and P-ERK. While gefitinib alone could significant inhibit the EGFR signaling pathway, including P-EGFR, P-ERK, and P-AKT, and could also inhibit the expression of CyclinD1, C-Myc, and Bcl-xl, but it had only slightly effect on the expression of YAP. The combination of simvastatin with gefitinib resulted in a significant inhibition of the EGFR signaling, including P-EGFR, P-ERK, and P-AKT, and in an augmented inhibition of CyclinD1, C-Myc, and Bcl-xl (Fig. 4g, h, i, Suppl. Fig. 5).

### The effect of simvastatin combined with sorafenib in HCC cells

**Fig. 5:**
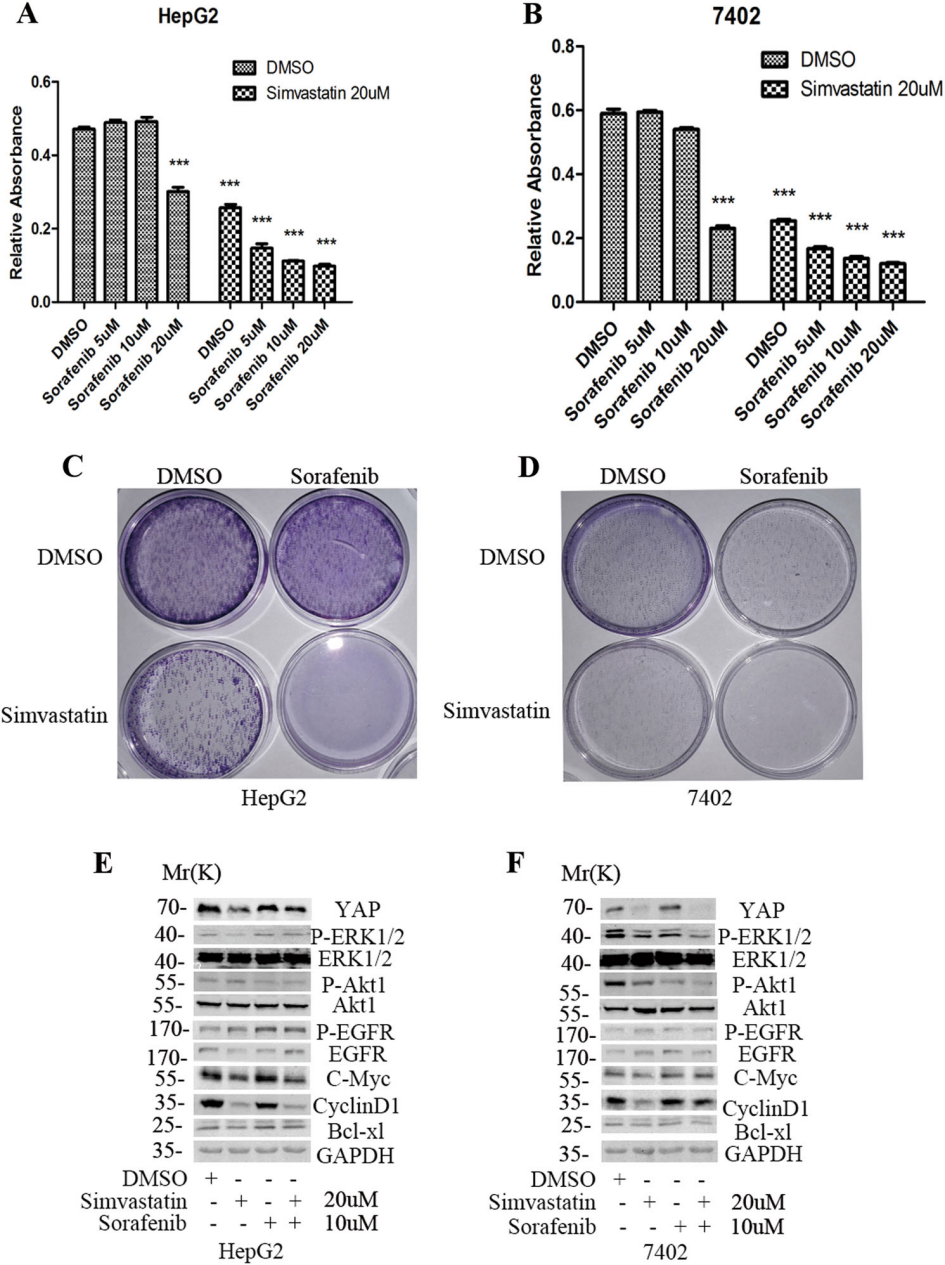
The combined effect of simvastatin and sorafenib in HCC cells. **a, b** CCK8 assays were used to detect the effect of simvastatin combined with sorafenib (Raf inhibitor) on the proliferation in HepG2 and SMMC7721 HCC cells. **c, d**, Colony formation assays were used to detect the effect of simvastatin combined with sorafenib on the survival of HCC cells. **e, f** WB was used to examine the effect of combined treatment on the core effectors of downstream signaling

Sorafenib, the only targeted drug used in HCC, is a multiple kinase inhibitor among its targets, including B-RAF and C-RAF. Sorafenib displays very limited extension of the survival of patients with advanced metastatic HCC, extending their life expectancy by 7.9 to 10.7 months^3^. So, it is urgent for us to find novel drugs that could enhance the cytotoxicity of sorafenib against HCC. Here, we combined simvastatin with sorafenib to simultaneously target YAP and RAF kinases in HCC cell lines. CCK8 assay indicated that simvastatin could significantly enhance the cytotoxicity of sorafenib in HepG2 and Bel7402 cells (Fig. 5a, b, Suppl. Table 1). The Dm value for simvastatin, sorafenib, and the combination treatment was 9.730, 7.844, and 9.448 μM, respectively, in HepG2 cells, and the combinations exhibited synergistic therapeutic interactions (CI < 1) across a wide range (~0.6–1) of Fa values (Suppl. Table 5, Suppl. Fig. 11). The Dm value for simvastatin, sorafenib, and the combination treatment was 11.715, 13.534, and 11.201 μM, respectively, in 7402 cells, and the combinations exhibited synergistic therapeutic interactions (CI < 1) across a wide range (~0–0.8) of Fa values (Suppl. Table 6, Suppl. Fig. 11). Colony formation assay indicated that the combined group inhibited the colony formation more significantly than that of simvastatin or sorafenib alone in the two HCC cell lines (Fig. 5c, d). Further mechanism study indicated that the combination of simvastatin with sorafenib resulted in a simultaneous inhibition of YAP, P-ERK, P-Akt1, and CyclinD1, C-Myc, and Bcl-xl in the three cell lines (Fig. 5e, f, Suppl. Fig. 6).

### The combination of simvastatin and trametinib in HCC cells

**Fig. 6:**
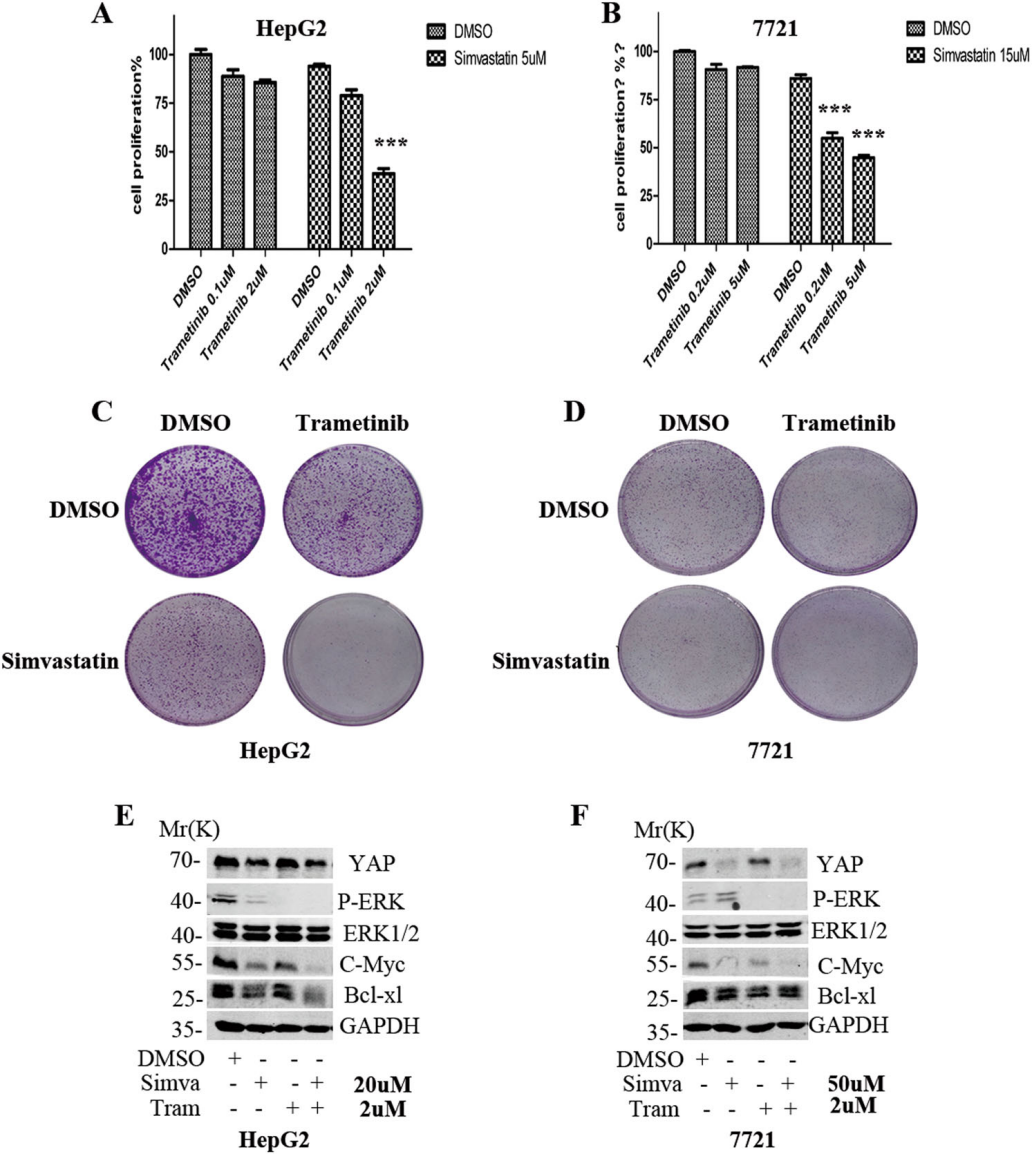
The combined effect of simvastatin and trametinib in HCC cells. **a, b** CCK8 assays were used to detect the effect of simvastatin combined with trametinib (MEK inhibitor) on the proliferation in HepG2 and SMMC7721 HCC cells. **c, d**, Colony formation assays were used to detect the effect of simvastatin combined with trametinib on the survival of HCC cells. **e, f** WB was used to examine the effect of combined treatment on the core effectors of downstream signaling

The RAS–RAF-MEK–MAPK pathway plays an important role in the carcinogenesis of diverse cancers^34^. Though the RAS proteins are undruggable at present, there has been successful drugs targeting RAF(vemurafenib, dabrafenib) and MEK(trametinib), and these have shown substantial clinical activity in melanoma^35–37^. Therefore, we also tested whether the YAP inhibitor simvastatin could potentiate the cytotoxic activity of MEK inhibitor trametinib in HCC. Our results indicated that trametinib alone could inhibit the proliferation of HCC cells, and that simvastatin could enhance the sensitivity of HCC cells to trametinib (Fig. 6a, b, Suppl. Table 1). The Dm value for simvastatin, trametinib, and the combination treatment was 10.629, 686.881, and 16.372 μM, respectively, in HepG2 cells, and the combinations exhibited synergistic therapeutic interactions (CI < 1) across a wide range(~0.1–0.8) of Fa values (Suppl. Table 7, Suppl. Fig. 12). The Dm value for simvastatin, trametinib, and the combination treatment was 64.313, 119.387, and 65.850 μM, respectively, in 7721 cells, and the combinations exhibited synergistic therapeutic interactions (CI < 1) across a wide range (~0.45–1) of Fa values (Suppl. Table 8, Suppl. Fig. 12). Colony formation assay indicated the combined treatment could induce a synergistic cytostatic effects on the HCC cells (Fig. 6c, d). WB results indicated that combined treatment could simultaneously inhibit the expression of YAP and P-ERK, and induce significantly down-regulation of C-Myc and Bcl-xl (Fig. 6e, f).

## Discussion

Recent studies have demonstrated that YAP, the core effector of the Hippo pathway, was involved in the carcinogenesis of many cancers, including HCC^5^. YAP activation is an early event in liver tumorigenesis, making which a potential therapeutic target for HCC treatment^14^. The dysregulated EGFR signaling also plays an important role in the carcinogenesis of HCC^19,20^. However, the crosstalk between the Hippo pathway and the EGFR signaling and its implication in target therapy is still poorly understood in HCC.

In the current study, we demonstrated that the EGFR signaling could bypass the canonical RhoA signaling and mainly act through PI3K-PDK1 pathway, but not the AKT or the MAPK pathway to activate YAP signaling. Notably, it has been reported that the EGF signaling inhibited the Hippo pathway through activation of PI3-kinase (PI3K) and phosphoinositide-dependent kinase (PDK1), other than that of AKT, thereby leading to inactivation of Lats, dephosphorylation of YAP, YAP nuclear accumulation and transcriptional activation of its target gene of CTGF in immortalized mammary cells^24^. At this point, our data was partly consistent with that published report. We found that EGF stimulation could enhance total YAP expression and promote its nuclear accumulation in HCC cells, whereas Run Fan et al. reported that the EGF treatment could lead to dephosphorylation of YAP and YAP nuclear accumulation, but had nearly no effect on total YAP expression^24^. The main reason for the difference might be the use of different cell types in the two studies. Our data also indicated that EGF stimulation could enhance the expression of Lats1, the upstream inhibitor of YAP, and that knockdown YAP could reverse EGF mediated up-regulation of Lats1 (Suppl. Fig. 13). Other studies showed that YAP/TAZ activation resulted in activation of LATS1/2, their upstream negative regulators, to constitute a negative feedback loop of the Hippo pathway in vitro and in vivo^38,39^. Together, the present data indicated that EGF induced Lats1 up-regulation might be the results of transcriptional induction of up-regulated YAP expression, thus YAP and Lats1 could also form a negative feed-back signaling loop in EGF-treated HCC cell, and the molecular mechanism still need further investigation.

We also found that the activated EGFR signaling could act through YAP to trans-activate Cyr61, one target gene of YAP. This result was similar to Raquel Urtasun et al.’s study which showed that the EGFR signaling could act through YAP to stimulate the expression of another target gene of YAP, CTGF in liver cancer, although, they didn’t illustrate the underlying mechanisms in details^25^. Other studies reported that the activated YAP and EGFR signaling could form a positive signaling loop to drive cancer progression in several cell types, including cervical cancer, esophageal cancer and breast epithelial cells^13,23,40^. However, in our study, we didn’t observe that YAP knockdown had any effect on the EGFR signaling. The difference in conclusions of these reports might be that the cell types used in these studies were so different.

The reported YAP inhibitors mainly include veteporfin and statins^28–30^. Several large population-based cohort studies in Asian and Western populations have shown a protective association between the use of statins and the risk of HCC^31,32^. Recent studies have indicated that statins could down-regulate YAP through inhibiting RhoA activity^29,30^. Here, we used simvastatin as a YAP inhibitor. Simvastatin could enhance the sensitivity of HCC cells to gefitinib, a EGFR specific TKI. Our results were consistent with a previous work demonstrating that atorvastatin could overcome gefitinib resistance in KRAS mutant human non-small cell lung carcinoma cells^41^.

The SHARP trial demonstrated that sorafenib could improve survival in patients with advanced metastatic HCC, extending life expectancy from 7.9 to 10.7 months^42^. Our study indicated that simvastatin could also enhance the cytotoxic activity of sorafenib, the only target drug used in HCC treatment. The targets of sorafenib includes B-Raf and C-Raf both of which act as the critical downstream effectors of EGFR signaling. Notably, one report has demonstrated that the combination of lovastatin and sorafenib produced synergistic cytotoxic effects against renal carcinoma cells^43^. We also investigated the effect of the combination of simvastatin with trametinib, the first FDA approved MEK inhibitor, on the cell proliferation and survival of HCC. Not surprisingly, the combined therapy produced synergistic lethality in HCC cells. At this point, our results were consistent with a very recent report which revealed that trametinib plus fluvastatin showed synergistic efficacy in both the drosophila Ras-Pten lung cancer model and human lung cancer cell line^44^.

Several recent studies indicated that YAP could serve as a novel biomarker for cetuximab resistance in colorectal cancer and head and neck cancer^12,45^, and our unpublished data showed that knockdown YAP could enhance the sensitivity of K-RAS mutant CRC cells to cetuximab. Notably, the YAP inhibitor, veteporfin and simvastatin, could overcome cetuximab resistance in K-RAS mutant CRC cells. Some studies showed that the activated YAP could bypass K-RAS to promote cell proliferation and survival in several tumor types, and that YAP could also promote resistance to RAF-targeted and MEK-targeted cancer therapies^46–48^. These findings indicated that the crosstalk between Hippo pathway and EGFR signaling played a vital role in the carcinogenesis of multiple malignancies, combined targeting YAP and the EGFR signaling might represent a novel therapeutic strategy for the treatment of HCC, as well as several other cancers (Suppl. Fig. 8).

**Fig. 7:**
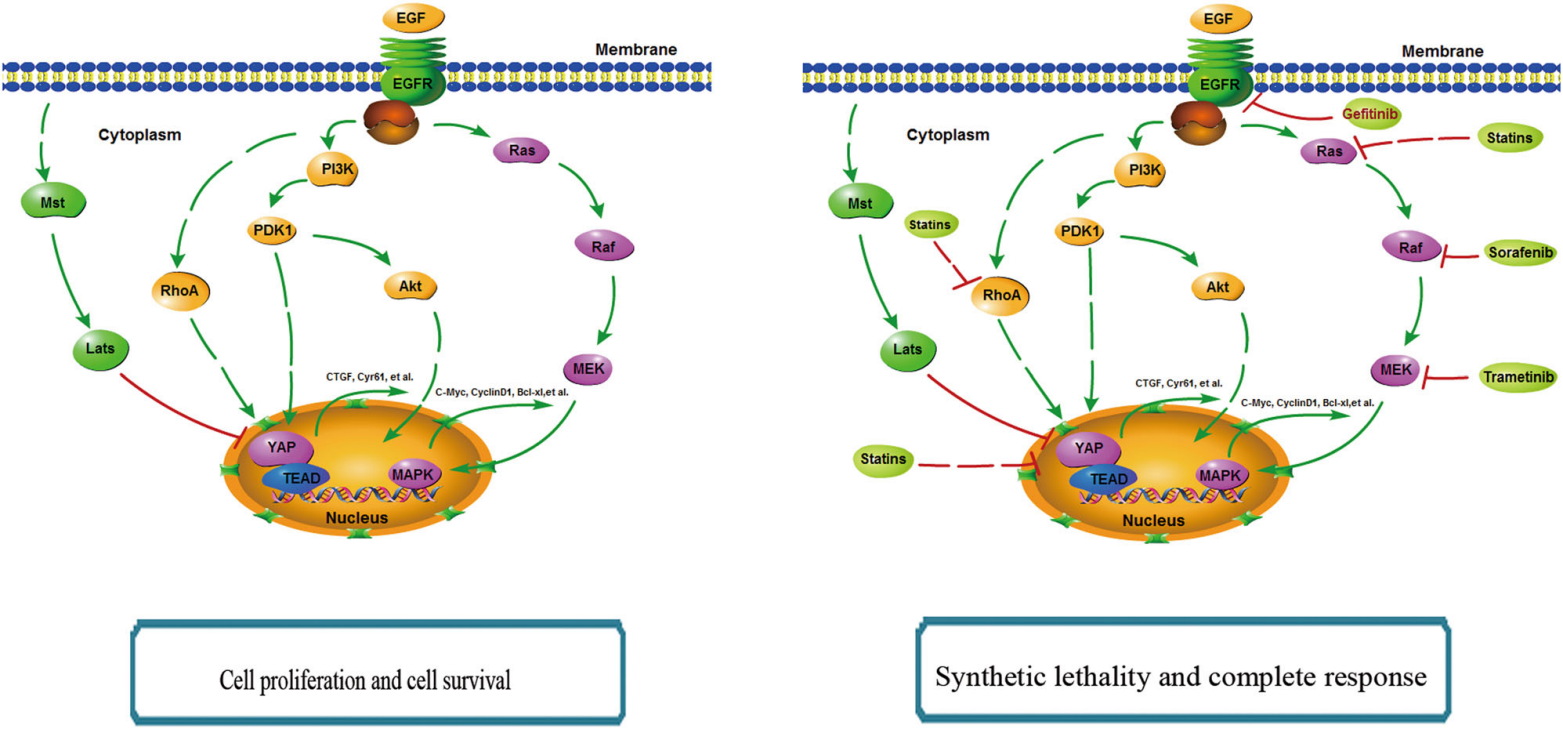
The proposed model of the regulation mechanisms of Hippo pathway by the EGFR signaling

In summary, we demonstrated that EGFR signaling could bypass RhoA and act mainly through the PI3K/PDK1 pathway to activate YAP. The activated EGFR signaling could promote the proliferation of HCC cells in a YAP-independent manner. Our findings also provided several promising poly-therapeutic strategies to enhance the efficacy of HCC treatment through combined targeting of YAP and the EGFR signaling (Fig. 7).

## Materials and methods

### Cell culture and the inhibitors

The human liver cell line HL7702, and the human HCC cell lines HepG2, Bel7402, Hep3B and SMMC7721 were from ATCC. All five cell lines were maintained in DMEM medium supplemented with 10% fetal bovine serum (HyClone, USA) in 5% CO_2_ at 37 °C. The inhibitors used in this study include the PI3K inhibitor wortamanin, PDK1 inhibitor GSK2334470 and BX-795, AKT inhibitor MK2206, MEK inhibitor Trametinib(Selleck, USA), EGFR inhibitors Gefitinib, PI3K inhibitor Ly294002, and MEK inhibitor U0126(MCE, USA).

### Western-blot

Cells were lysed in RIPA buffer (150 mM NaCl, 1% NP-40, 50 mM Tris-HCl PH 7.4, 1 mM phenylmethylsulfonyl fluoride, 1 μg/ml leupeptin, 1 mM deoxycholic acid and 1 mM EDTA) containing a cocktail of protease inhibitors and phosphatase inhibitors (Calbiochem, Darmstadt, Germany). Equal amounts of protein sample (30–50 μg) were separated by 12% SDS-PAGE and transferred to NC membrane (Millipore, Bedford, MA, USA) using the Bio-Rad wet transfer system. The following antibodies were used for Western blotting: Mst1, YAP, P-YAP(ser127), P-EGFR(1068), P-ERK1/2(202/204), ERK1, P-AKT(ser397), AKT1, C-Myc, CyclinD1 (Epitomics, USA); YAP/TAZ, Lats1, Bcl-xl (CST, USA), EGFR, RhoA (Santa Cruze, USA), Cyr61 (Proteintech, China), GAPDH and β-actin (Biostar, China).

### Cytoplasmic and nuclear protein extraction

The serum-starved(one night) HepG2 and 7721 cells were stimulated with EGF(20 ng/ml) for 2 h, then the cytoplasmic and nuclear proteins were extracted using Keygen Nuclear and Cytoplasmic Extraction Kit (Keygen Biotec, Nanjing, China) according to the manufacturer’s protocol.

### Pull-down assays

Active RhoA in cell lysates (200 μg) was precipitated with 15 μg GST-RBD (containing amino acids-8–89 of Rhotekin), which was expressed in Escherichia coli and bound to agarose beads. The precipitates were washed three times in washing buffer [50 mmol/L Tris(pH 7.2), 150 mmol/L NaCl, 10 mmol/L MgCl2, 0.1 mmol/Lphenylmethylsulfonyl fluoride, 10 μg/ml aprotinin, and 10 μg/ml leupeptin], and after adding the loading buffer and boiling for 5 min, the bound proteins were resolved in 12% polyacrylamide gels, transferred to NC membranes, and immunoblotted with anti-RhoA antibody as described above.

### Fluorescence microscopy

HepG2 and SMMC7721 cells were stimulated by serum-free 20 ng/ml EGF for 4 h before plating into 24-well dishes containing 12 mm glass coverslips. Then cells were fixed by 4% paraformaldehyde for 20 min and incubated in blocking buffer for another 1 h (0.5% triton X-100 and 1% BSA in PBS) prior to primary antibody staining. Both primary and secondary antibodies were diluted in blocking buffer. Coverslips were incubated with primary antibodies for 1 h at room temperature followed by 1 h second antibodies incubation. In the end, diluted DAPI was added on the coverslips, 10 min later, coverslips were mounted on the glass slide. Images were captured under Nikon Eclipse 80i (Nikon, Japan) microscope with NIS-Elements software (version 4.30.01).

### Cell transfection

The SiRNAs against RhoA and YAP were transfected into gastric cancer cells, respectively, by Lipofectamine 2000 (Invitrogen, USA), and the non-sense RNA was used as a negative control. The sequence of the two siRNAs were presented in our previous study^17^.

### Cell viability assay

HCC cells were seeded in 96-well plates for one night, then cells were transfected with siRNA or negative control, respectively, for 24 h, next, the cells were treated by different drugs or the combined therapy. Cell growth was measured by Cell Counting Kit-8 (Dojingdo, Kumamoto, Japan). The coefficient of drug interaction (CDI) was used to analyze the synergistically inhibitory effect of drug combinations^49^. CDI is calculated as follows: CDI = AB/(A*B). According to the absorbance of each group, AB is the ratio of the combination groups to the control group; A or B is the ratio of the single agent groups to the control group. Thus a CDI value less than, equal to or greater than 1 indicates that the drugs are synergistic, additive or antagonistic, respectively. CDI less than 0.7 indicate that the drugs are significantly synergistic.

### Colony formation

The 4000–5000 cells were seeded in 3 cm dish for one night, then the cells were incubated with different inhibitors for 4–6 days. Next, cells were washed by PBS and fixed by 4% paraformaldehyde for 20 min, further crystal violet staining was used to observe the effect of treatment on the survival of HCC cells.

### Statistical analysis

The qualification of WB was performed using the Image J. All of the statistical calculations were performed using the GraphPad Prism 5. The data were expressed throughout as means ± the standard error of the mean (SEM). Statistical comparisons were performed by one-way ANOVA. Tukey’s post hoc test was used for multiple group comparisons and Student’s *t* test was used for single comparisons. All of the *p*-values were two-sided and *p* < 0.05 was considered to be statistically significant.

### Availability of data and materials

Data and materials will be shared.

## Electronic supplementary material

Suppl. inform
