# Supplementary material for: EGFR-PI3K-PDK1 pathway regulates YAP signaling in hepatocellular carcinoma: the mechanism and its implications in targeted therapy
Source: Cell Death Dis. 2018 Feb 15;9(3):269. doi: 10.1038/s41419-018-0302-x (PMC5833379; doi:10.1038/s41419-018-0302-x)
Supplement: Supplementary file 1 — Suppl. inform [file 41419_2018_302_MOESM1_ESM.docx]

**Additional supplementary materials**

**EGFR-PI3K-PDK1 pathway regulates YAP signaling in hepatocellular carcinoma: the mechanism and its implication in targeted therapy**

Hongwei Xia^1&^, Xinyu Dai^2&^, Huangfei Yu^2&^, Sheng Zhou^2^, Zhenghai Fan^2^, Bisheng Liu^2^, Guoqing Wei^1^, Huanji Xu^2^, Weibing Leng^2^, Qing Liu^2^, Qiulin Tang^1^, Qiyong Gong ^3^, Feng Bi ^1,2*^

1 Laboratory of Molecular Targeted Therapy of Oncology, State Key Laboratory of Biotherapy and Cancer Center, West China Hospital, Sichuan University, and Collaborative Innovation Center for Biotherapy. 2 Department of Medical Oncology, West China Hospital, Sichuan University, Chengdu, Sichuan Province, 610041, China. 3 Department of Radiology, West China Hospital of Sichuan University, Chengdu, Sichuan Province, 610041, China.

**
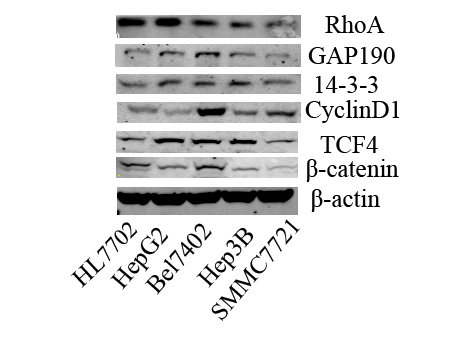
**

**Supplementary Fig. 1. The expression of the core members of Wnt pathway and RhoA signaling in HCC cells**

WB was used to examine the expression of the core members of Wnt pathway and RhoA signaling in HCC cells.

**
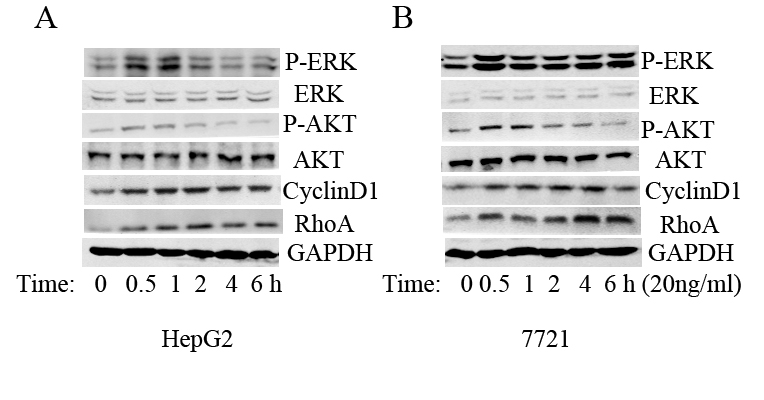
**

**Supplementary Fig. 2. The effect of EGF on the expression of EGFR pathway and RhoA.**

WB was used to study the effect of EGF stimulation on the the core members of EGFR pathway, CyclinD1 and RhoA at different time point in HepG2 (A)and SMMC7721 (B) cells.

**
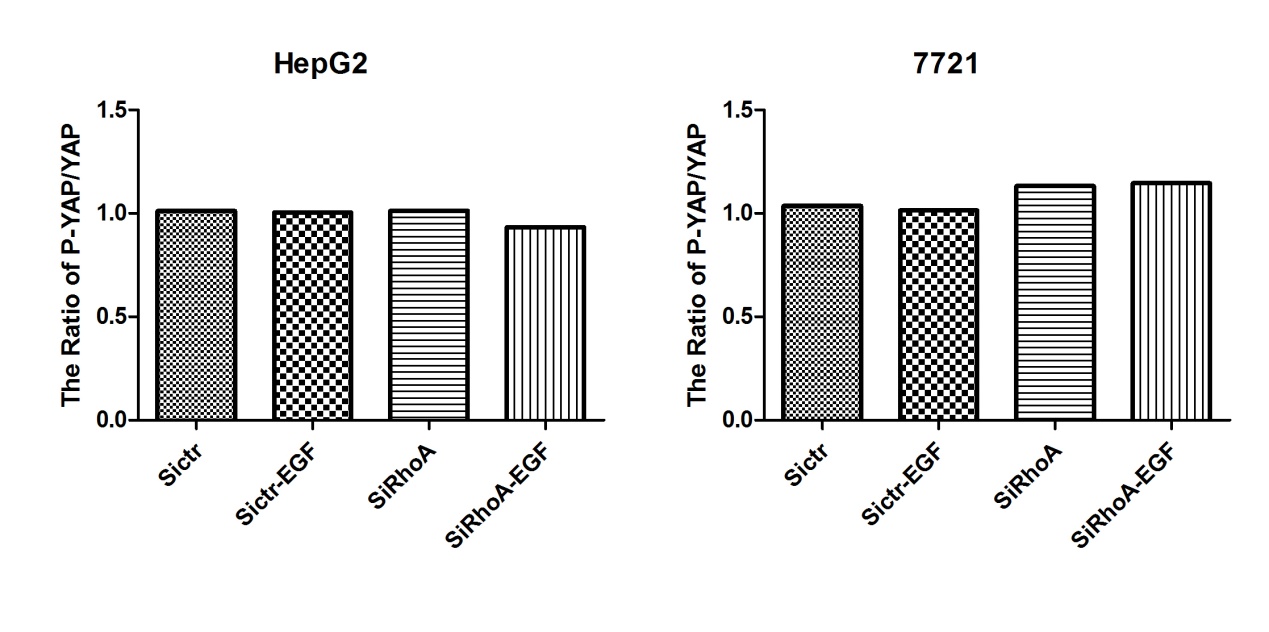
**

**Supplementary Fig. 3. The effect of EGF treatment and SiRhoA on the phosphorylational level of YAP.**

The band of WB (Fig 2C,D) was quantified by the Image J.


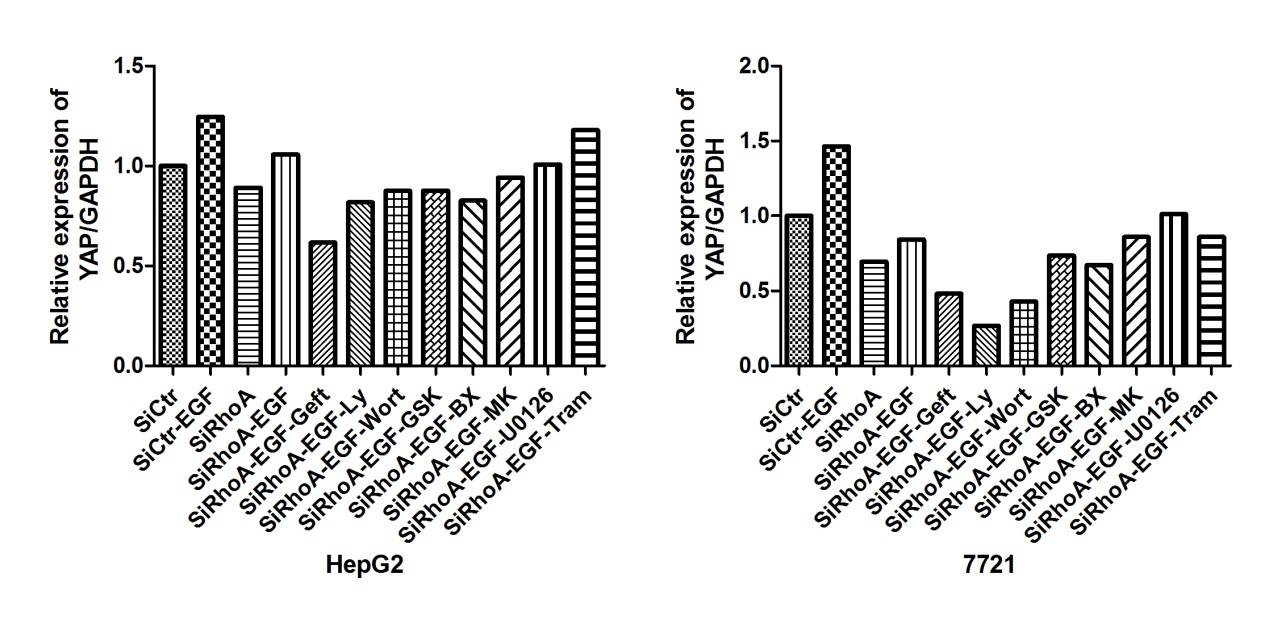


**Supplementary Fig. 4. The relative YAP expression(YAP/GAPDH) in Fig 3 A,D**

The WB of Fig 3 A, D was quantified using the Image J.


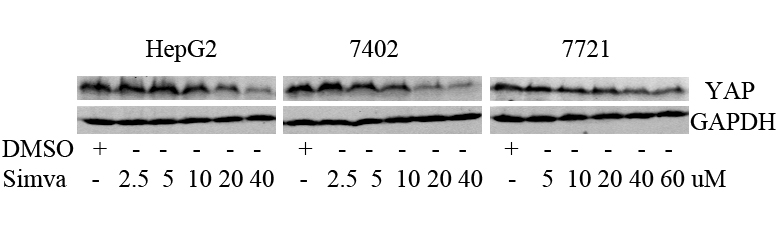


**Supplementary Fig. 5. The effect of simvastatin treatment on the expression of YAP.**

Three HCC cells were treated with different concentrations of simvastatin for 24hours, then WB was used to examine the expression of YAP in these cells. GAPDH was used as loading controls.


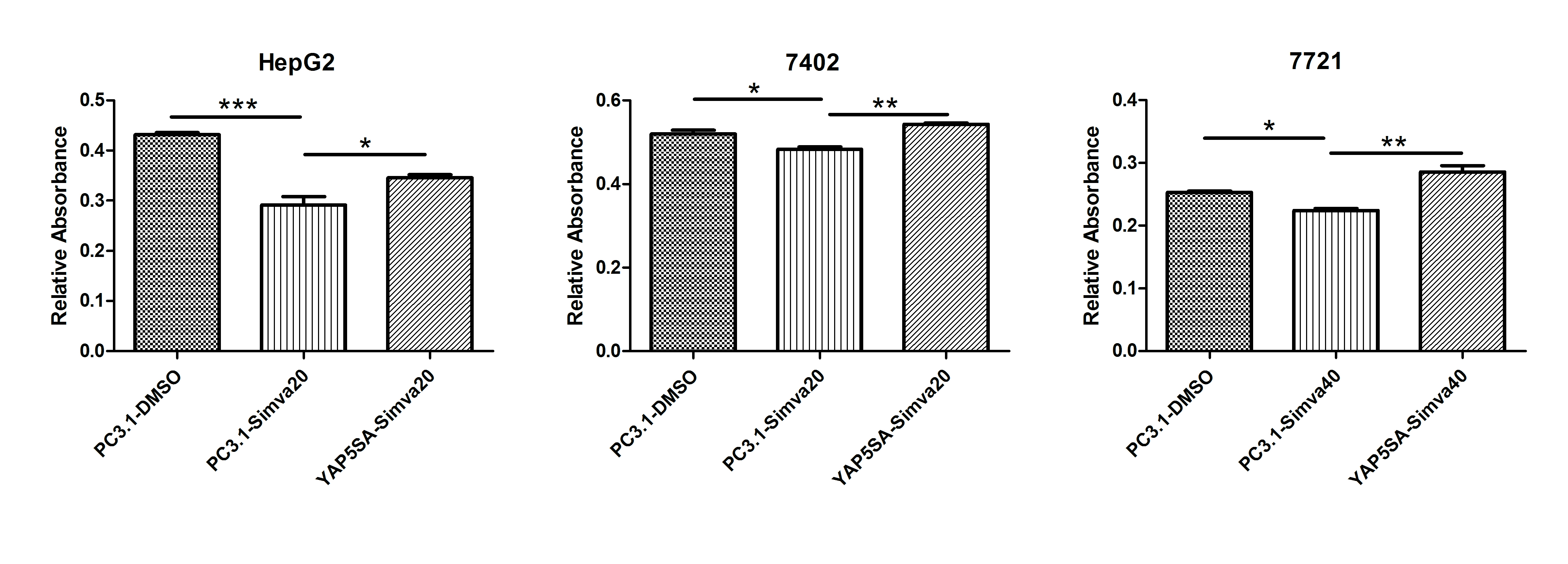


**Supplementary Fig. 6. Forced expression of YAP could reverse the inhibition of cell proliferation induced by simvastatin.** The HCC cells were plated in the 96 well plates, then cells were transfected with PCDNA 3.1 or the YAP5SA( the active YAP) plasmid, 24 hours later, the transfected cells were treated with DMSO or the simvastatin for 48hours, CCK8 was used to detect the cell viability. T-test was used to detect the difference.* P<0.05, ** P<0.01, *** P<0.001.


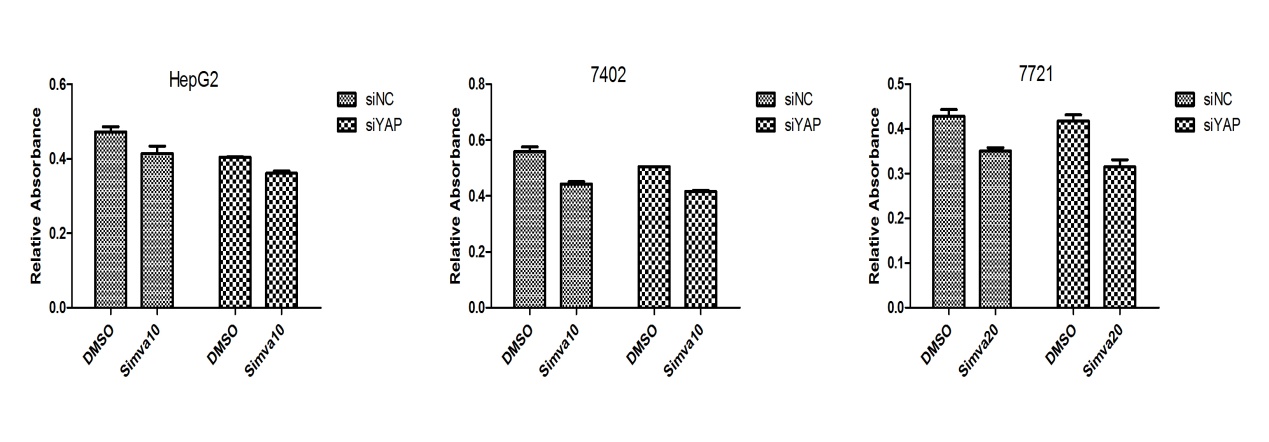


**Supplementary Fig. 7. YAP down-regulation combined with simvastatin in HCC cells.** The HCC cells were plated in the 96 well plates, then cells were transfected with negative si-NC (siRNA controls) or the Si-YAP, 24 hours later, the transfected cells were treated with DMSO or the simvastatin for 48hours, CCK8 was used to detect the cell viability.


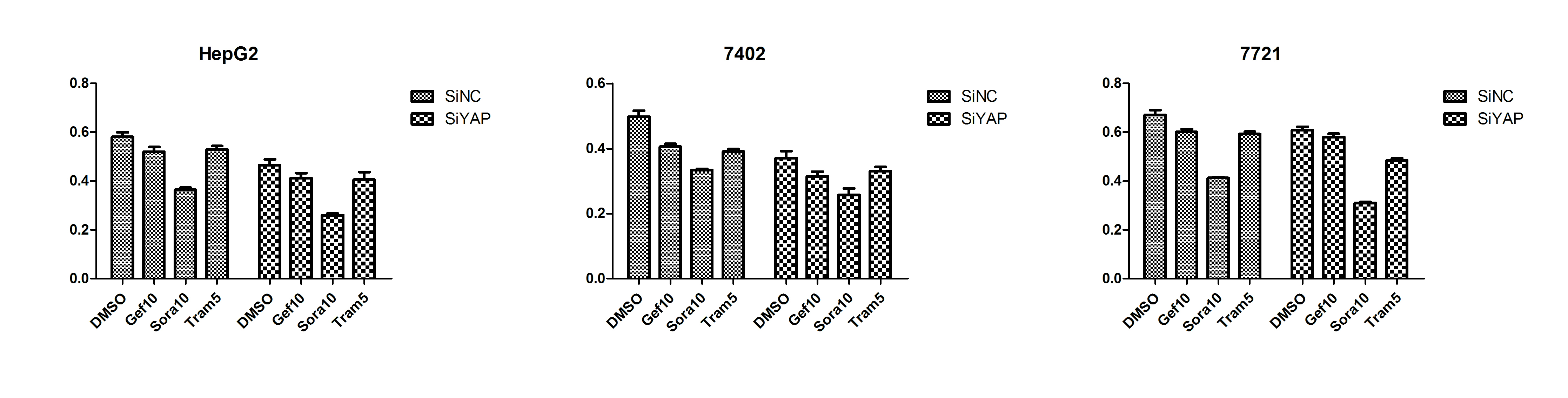


**Supplementary Fig. 8. YAP down-regulation could enhance the cytotoxicities of the inhibitors against the HCC cells.**

The HCC cells were transfected with the scramble SiRNAs or the YAP siRNAs, 24 hours later, cells were treated with different inhibitors for 48hours, CCK8 was used to detect the cell viability.


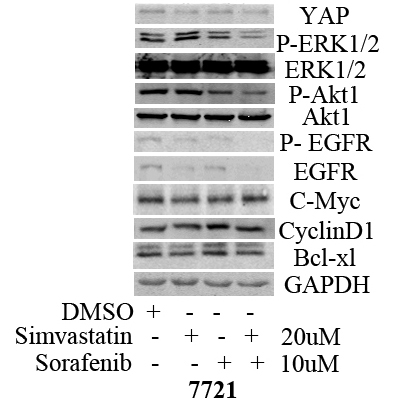


**Supplementary Fig. 9 The combined effect of simvastatin and sorafenib on the EGFR signaling in SMMC7721 cells.**

SMMC7721 cells were treated with simvastatin and sorafenib for 48hrs, then WB was used to examine the effect of combined treatment on the core effectors of downstream signaling.

**
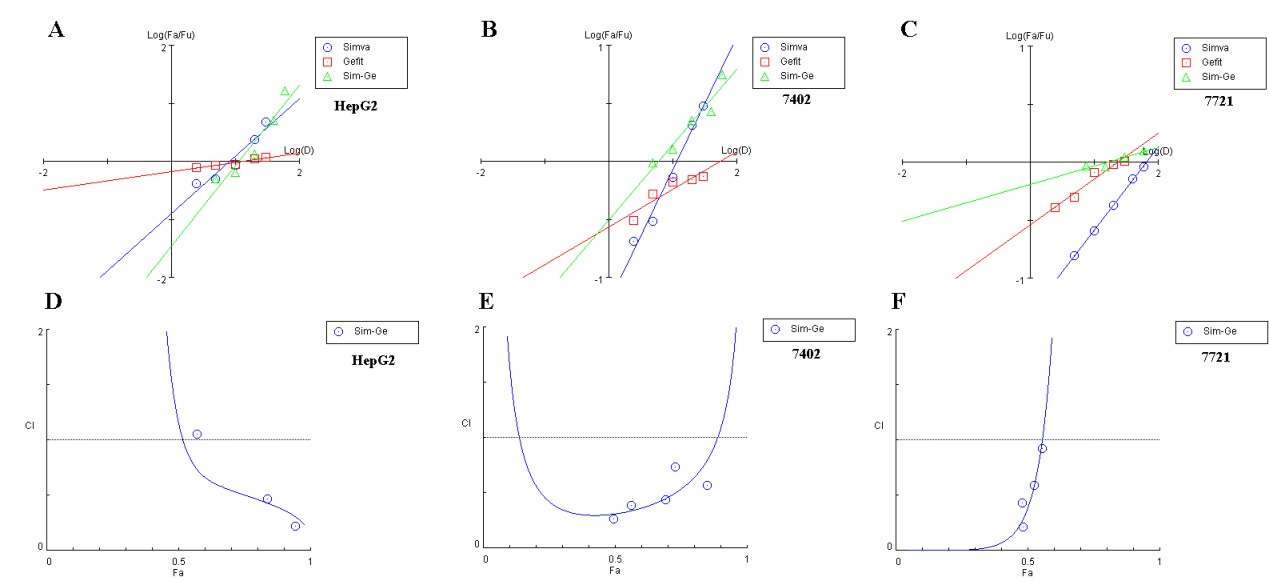
**

**Supplementary Fig. 10. The results of the median effect analysis for the interaction of simvastatin and gefitinib in the three HCC cells.** A, B, C The median effect plot for the interaction of simvastatin and gefitinib in HepG2, 7402 and 7721 cells following 48 hrs drug exposure. And the cells were treated with simvastatin(○), gefitinib(□), and simvastatin and gefitinib a 1:1 molar ratio(△) . Fa = the fraction of HCC cells affected by the drugs, Fu = the fraction of cells unaffected, and D = drug dose. D, E, F Combination index analysis of the interaction of simvastatin and gefitinib in the three HCCcells following 48 hrs of treatment. ○ Indicate where doses studied fall on the combination index curve.  **Abbreviations:** Simvastatin: simva, Sim; Gefitinib: Gefit, Ge.

**
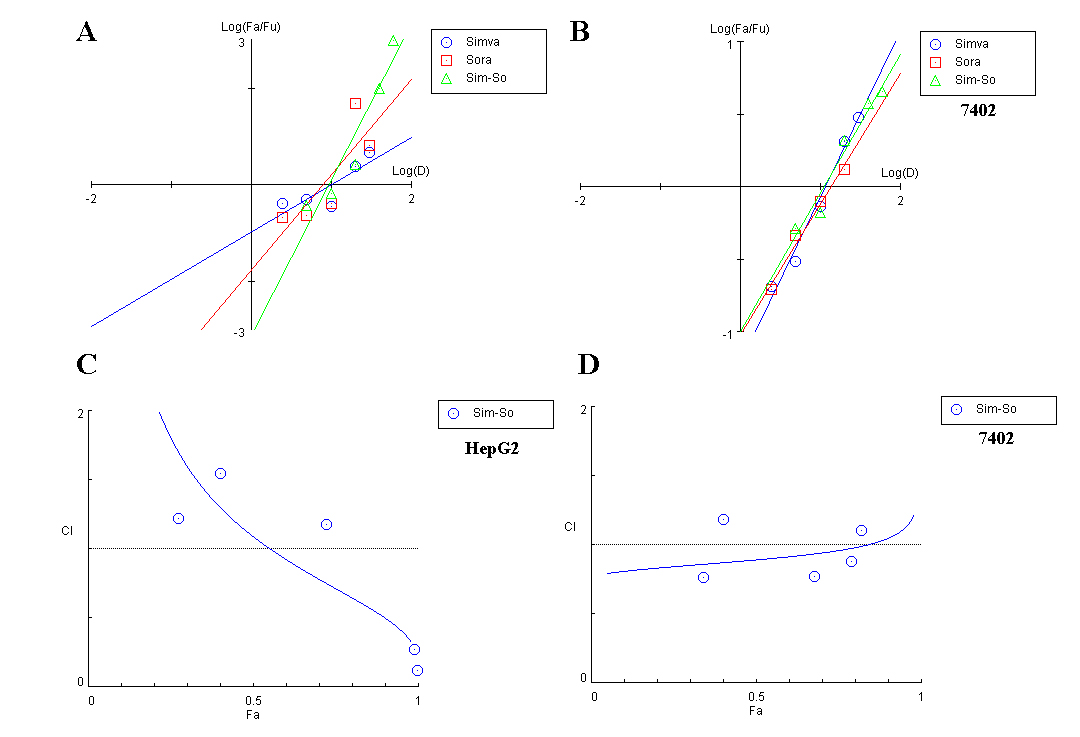
**

**Supplementary Fig. 11 The results of the median effect analysis for the interaction of simvastatin and sorafenib in the three HCC cells.** A, B, C The median effect plot for the interaction of simvastatin and sorafenib in HepG2, and 7402 cells following 48 hrs drug exposure. And the cells were treated with simvastatin(○), sorafenib (□), and simvastatin and sorafenib in a 1:1 molar ratio(△) . Fa = the fraction of HCC cells affected by the drugs, Fu = the fraction of cells unaffected, and D = drug dose. D, E, F Combination index analysis of the interaction of simvastatin and gefitinib in the three HCC cells following 48 hrs of treatment. ○ Indicate where doses studied fall on the combination index curve. **Abbreviations:** Simvastatin: simva, Sim; Sorafenib: Sora, So.


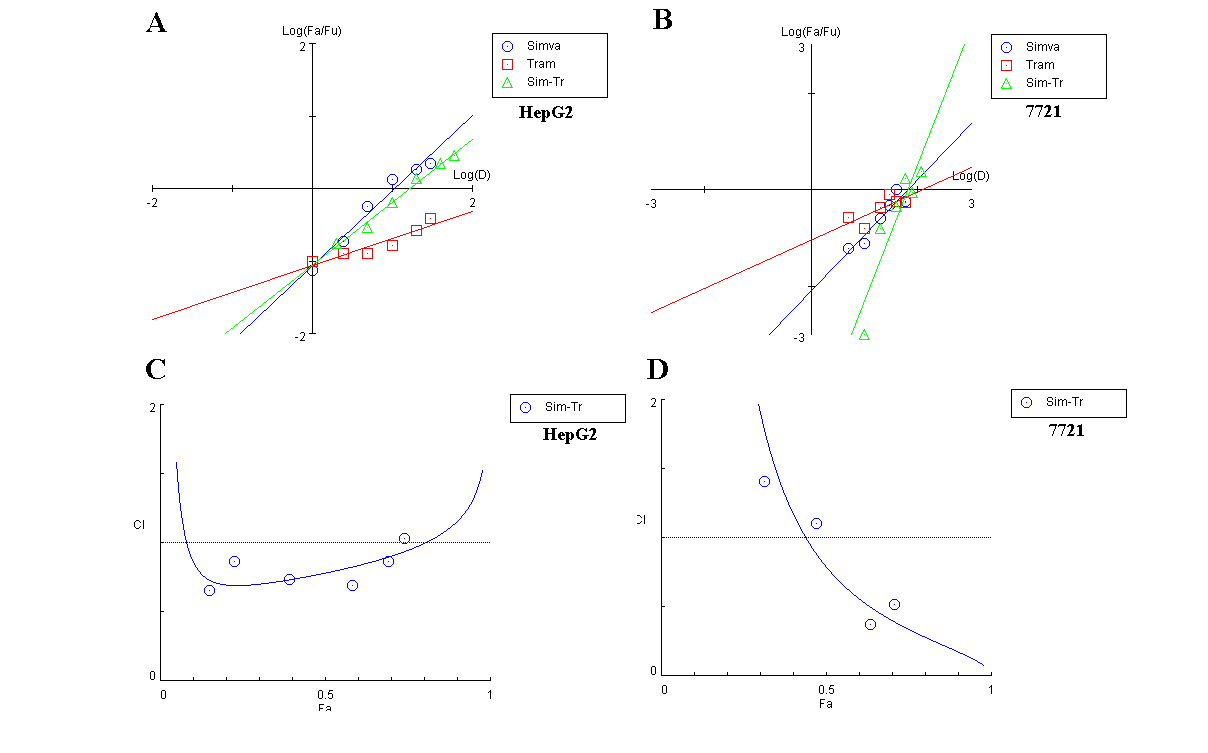


**Supplementary Fig. 12 The results of the median effect analysis for the interaction of simvastatin and sorafenib in the three HCC cells.** A, B, C The median effect plot for the interaction of simvastatin and trametinib in HepG2, and 7721 cells following 48 hrs drug exposure. And the cells were treated with simvastatin(○), trametinib (□), and simvastatin and trametinib in a 1:1 molar ratio(△) . Fa = the fraction of HCC cells affected by the drugs, Fu = the fraction of cells unaffected, and D = drug dose. D, E, F Combination index analysis of the interaction of simvastatin and trametinib in the two HCC cells following 48 hrs of treatment. ○ Indicate where doses studied fall on the combination index curve.  **Abbreviations**: Simvastatin: simva, Sim; Trametinib: Tram, Tr.

**
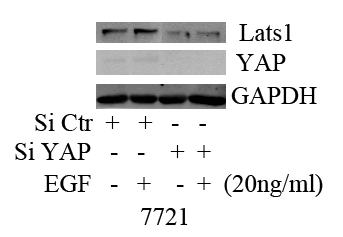
**

**Supplementary Fig. 13. EGF acts through YAP to regulate Lats1 in HCC cells.**

A, B The Si-YAP was transfected into cell for 48hours in SMMC7721, then the cells were incubated with the serum-free medium for one night, further 20ng/ml EGF was added into the serum-free medium for another 4hours. WB was used to detect the effect of EGF combined with YAP knockdown on the expression of Lats1 in the two HCC cell lines.

**Supplementary. Table 1. The coefﬁcient of drug interactions**

| **Cell** |  | Simva 20uM | |  | Simva 20uM | | Simva 5uM | Simva  15uM | Simva  5uM | | Simva  15uM |
| --- | --- | --- | --- | --- | --- | --- | --- | --- | --- | --- | --- |
|  | Gefi 5uM | Gefi 10uM | Gefi 20uM | Sora 5uM | Sora 10uM | Sora 20uM | Tram 50nM | Tram 200nM | Tram  2uM | | Tram 5uM |
| **HepG2** | 1.09 | **0.96** | **0.97** | 1.17 | **0.88** | 1.268 | **0.95** |  | **0.48** |  | |
| **7402** | 1.26 | **0.99** | 1.189 | 1.1 | **0.99** | 2.048 |  |  |  | |  |
| **7721** | 1.37 | 1.36 | **0.66** |  |  |  |  | **0.71** |  | | **0.57** |

CDI = AB/(A*B). According to the absorbance of each group, AB is the ratio of the combination groups to control group; A or B is the ratio of the single agent groups to control group. Thus a coefﬁcient of drug interaction (CDI) value less than, equal to or greater than 1 indicates that the drugs are synergistic, additive or antagonistic, respectively. CDI less than 0.7 indicate that the drugs are signiﬁcantly synergistic. Simva, Simvastatin; Gefi, Gefitinib; Sora, Sorafenib; Tram, Trametinib.

**Supplementary. Table 2 Calculated values for the Dm and Combination Index as a function of fractional inhibition of HepG2 cell proliferation by a mixture of Simvastatin and Gefitinib.**

| **HepG2**  **Drug/Combo** | **Parameters** | | | **CI values at:** | | | | |
| --- | --- | --- | --- | --- | --- | --- | --- | --- |
|  | **Dm** | **m** | **r** | **ED55** | **ED75** | **ED90** | **ED95** | |
| Simva | 8.140 | 0.992 | 0.961 |  | | | |  |
| Gefit | 12.183 | 0.161 | 0.964 |  |  |  |  |  |
| Simva-Gefit | 11.212 | 1.392 | 0.954 | 0.802 | 0.502 | 0.365 | 0.294 | |

Dm is the dose required to produce the median effect (analogous to the IC50), and m is the Hill coefficient used to determine whether the dose effect relationships follow sigmoidal dose-response curves (Hill, 1913). Linear regression correlation coefficients (r-values) of the median effect plots reflect that the dose-effect relationships for Simvastatin, Gefitinib, and the combination, con-form to the principle of mass action (in general, r values>0.9 confirm the validity of this methodology) . CI is the Combination Index. **Abbreviations**: Simva, Simvastatin; Gefit, Gefitinib.

**Supplementary. Table. 3 Calculated values for the Dm and Combination Index as a function of fractional inhibition of 7402 cell proliferation by a mixture of Simvastatin and Gefitinib.**

| **7402**  **Drug/Combo** | **Parameters** | | | **CI values at:** | | | | |
| --- | --- | --- | --- | --- | --- | --- | --- | --- |
|  | **Dm** | **m** | **r** | **ED15** | **ED25** | **ED50** | **ED75** | |
| Simva | 11.715 | 1.146 | 0.990 |  | | | |  |
| Gefit | 53.759 | 0.327 | 0.921 |  |  |  |  |  |
| Simva-Gefit | 5.962 | 0.652 | 0.965 | 0.863 | 0.420 | 0.310 | 0.536 | |

**Supplementary. Table. 4 Calculated values for the Dm and Combination Index as a function of fractional inhibition of 7721 cell proliferation by a mixture of Simvastatin and Gefitinib**.

| **7721**  **Drug/Combo** | **Parameters** | | | **CI values at:** | | | | |
| --- | --- | --- | --- | --- | --- | --- | --- | --- |
|  | **Dm** | **m** | **r** | **ED10** | **ED30** | **ED50** | **ED55** | |
| Simva | 65.086 | 0.722 | 0.999 |  | | | |  |
| Gefit | 23.480 | 0.396 | 0.975 |  |  |  |  |  |
| Simva-Gefit | 15.813 | 0.157 | 0.941 | 5.33E-5 | 0.011 | 0.386 | 0.922 | |

**Supplementary. Table. 5 Calculated values for the Dm and Combination Index as a function of fractional inhibition of HepG2 cell proliferation by a mixture of Simvastatin and Sorafenib.**

| **HepG2**  **Drug/Combo** | **Parameters** | | | **CI values at:** | | | |
| --- | --- | --- | --- | --- | --- | --- | --- |
|  | **Dm** | **m** | **r** | **ED60** | **ED75** | **ED90** | **ED95** |
| Simva | 9.730 | 0.979 | 0.844 |  | | | |
| Sora | 7.844 | 1.984 | 0.826 |  |  |  |  |
| Simva-Sora | 9.448 | 3.202 | 0.950 | 0.921 | 0.711 | 0.498 | 0.403 |

**Abbreviations**: Simva, Simvastatin; Sora, Sorafenib.

**Supplementary. Table. 6 Calculated values for the Dm and Combination Index as a function of fractional inhibition of 7402 cell proliferation by a mixture of Simvastatin and Sorafenib.**

| **7402**  **Drug/Combo** | **Parameters** | | | **CI values at:** | | | | |
| --- | --- | --- | --- | --- | --- | --- | --- | --- |
|  | **Dm** | **m** | **r** | **ED10** | **ED30** | **ED50** | **ED80** | |
| Simva | 11.715 | 1.146 | 0.990 |  | | | |  |
| Sora | 13.534 | 0.904 | 0.991 |  |  |  |  |  |
| Simva-Sora | 11.201 | 0.961 | 0.980 | 0.809 | 0.852 | 0.892 | 0.98 | |

**Supplementary. Table. 7 Calculated values for the Dm and Combination Index as a function of fractional inhibition of HepG2 cell proliferation by a mixture of Simvastatin and Trametinib.**

| **HepG2**  **Drug/Combo** | **Parameters** | | | **CI values at:** | | | | |
| --- | --- | --- | --- | --- | --- | --- | --- | --- |
|  | **Dm** | **m** | **r** | **ED10** | **ED30** | **ED50** | **ED80** | |
| Simva | 10.629 | 1.041 | 0.983 |  | | | |  |
| Tram | 686.881 | 0.373 | 0.938 |  |  |  |  |  |
| Simva-Tram | 16.372 | 0.872 | 0.994 | 0.858 | 0.702 | 0.782 | 0.997 | |

**Abbreviations**: Simva, Simvastatin; Tram, Trametinib.

**Supplementary. Table 8 Calculated values for the Dm and Combination Index as a function of fractional inhibition of 7721 cell proliferation by a mixture of Simvastatin and Trametinib.**

| **7721**  **Drug/Combo** | **Parameters** | | | **CI values at:** | | | |
| --- | --- | --- | --- | --- | --- | --- | --- |
|  | **Dm** | **m** | **r** | **ED50** | **ED75** | **ED90** | **ED95** |
| Simva | 64.313 | 1.157 | 0.939 |  | | | |
| Tram | 119.387 | 0.501 | 0.768 |  |  |  |  |
| Simva-Tram | 65.850 | 2.850 | 0.912 | 0.788 | 0.336 | 0.173 | 0.115 |
